# First-in-human study to assess the safety, tolerability, pharmacokinetics and immunogenicity of DS002, an anti-nerve growth factor monoclonal antibody

**DOI:** 10.3389/fphar.2022.1075309

**Published:** 2022-12-12

**Authors:** Tingting Ma, Bei Cao, Lei Huang, Yuanxun Yang, Yan Geng, Pinhao Xie, Yu Zhao, Hui Lin, Kun Wang, Chunhe Wang, Runbin Sun, Juan Li

**Affiliations:** ^1^ Phase I clinical Trials Unit, Nanjing Drum Tower Hospital, The Affiliated Hospital of Nanjing University Medical School, Nanjing, China; ^2^ Beijing Highthink Pharmaceutical Technology Service Co., Ltd., Beijing, China; ^3^ Dartsbio Pharmaceuticals Ltd., Zhongshan, Guangdong, China

**Keywords:** DS002, anti-nerve growth factor antibody, first-in-human trial, pharmacodynamics, novel analgesic

## Abstract

**Purpose:** To evaluate the safety, tolerability, pharmacokinetics and immunogenicity of DS002 injection, an anti-nerve growth factor (anti-NGF) monoclonal antibody for treating pain conditions, in healthy Chinese subjects.

**Methods:** This study was a single-center, randomized, double-blind, single-dose escalation, placebo-controlled design (CTR20210155). A total of 53 healthy subjects, 27 male and 26 female, were enrolled in this study, and one subject withdrew from the study before administration. Seven dose groups were set up, which were 0.5 mg, 1.0 mg, 2.0 mg, 4.0 mg, 7.0 mg, 12.0 mg and 20.0 mg, respectively. The drug was administered by single subcutaneous injection. Four subjects were enrolled in the first dose group (0.5 mg) received DS002. Other dose groups enrolled eight subjects each, six of whom received DS002 while the other two received a placebo. Safety, tolerability, pharmacokinetic parameters and immunogenicity of DS002 were assessed.

**Results:** DS002 was well tolerated; all adverse events were Grade 1–2, and did not reach the termination standard of dose increment within the range of 0.5–20.0 mg. Adverse event rates were generally similar across treatments. After a single subcutaneous injection, the median T_max_ in different dose groups ranged 167.77–337.38 h; mean t_1/2_ ranged 176.80–294.23 h, the volume of distribution (V_z_) ranged 5265.42–7212.00 ml, and the clearance rate (CL) ranged 12.69–24.75 ml/h. In the dose range of 0.5–20.0 mg, C_max_ ranged from 51.83 ± 22.74 ng/ml to 2048.86 ± 564.78 ng/ml, AUC_0-t_ ranged from 20615.16 ± 5698.28 h·ng/mL to 1669608.11 ± 387246.36 h·ng/mL, and AUC_0-inf_ ranged from 21852.45 ± 5920.21 h·ng/mL to 1673504.66 ± 389106.13 h·ng/mL. They all increased with dose escalation, and C_max_ and AUC_0-t_ did not have a significant dose-linear relationship, whilst AUC_0-t_ was not dose-dependent at all. anti-drug antibody test results of each group of all subjects in this trial were negative.

**Conclusion:** DS002 showed satisfactory safety within the dose range of 0.5 mg–20.0 mg. The absorption and metabolism of DS002 were slow, it exhibited a low volume of distribution and the clearance rate was low. These data suggest that DS002, by blocking nerve growth factor, is expected to become a novel, safe and non-addictive treatment for pain conditions.

## Introduction

Chronic pain affects more than 30% of people in the world. The individual and societal burden of chronic pain are substantial (Cohen et al., 2021). In the state of chronic pain, the protective effect of pain becomes disordered and morbid. The pain attack leads to sleep disorder, anorexia, mental collapse, personality distortion and other consequences. Many patients even choose to commit suicide because they can’t bear the long-term pain, which jeopardized their lives and quality of life (Hooley et al., 2014). Non-steroidal anti-inflammatory drugs (NSAIDs) and opioid analgesics are the traditional main treatment for chronic pain (Chang et al., 2016). However, long-term use of NSAIDs causes adverse effects related to gastric bleeding and cardiovascular (Schjerning et al., 2020). Opioids are effective short-term treatment but have addictive and respiratory inhibitory effects (Coussens et al., 2019; Kiyatkin 2019), which limits their clinical application. Therefore, the successful management of chronic pain remains a significant medical challenge.

The nerve growth factor (NGF) belongs to the neurotrophic factor family. It was originally extracted from mouse submandibular gland and snake venom, and exists in almost all vertebrates (Tong et al., 2012). NGF exists in various species as different polymers (precursors), among which the β subunit has NGF biological activity and is called β-NGF; Mature dissociation β-NGF is composed of two 118-amino acid peptides connected through non-covalent bonds (Shooter 2001). Although NGF has a critical role in early neural development, in adults, this role changes to other functions, including neuronal plasticity, hypersensitization to noxious stimuli, and pain signaling. (Enomoto et al., 2019; Wise et al., 2021). When NGF binds to the functional receptor tropomyosin receptor kinase A (TrkA) on the nociceptor, it activates cytoplasmic ERK, PLC/PKC and other signal pathways. NGF-TrkA signaling reduces the threshold of the neuronal action potential, improves neuronal excitability, and then sensitizes pain (Tong et al., 2012; Wise et al., 2021). Elevated NGF levels have been associated with acute and chronic pain conditions and injured and inflamed tissues. NGF is expected to become a potential non-addictive analgesic target. The anti-NGF antibody can bind to NGF and block its interaction with TrkA to interrupt the pain-sensing neurons to send signals.

Anti-NGF antibody drugs include Pfizer/Lilly’s Tanezumab and Regeneron/Tiva’s Fasinumab, both of which have achieved significant efficacy in the treatment of moderate to severe osteoarthritis and chronic lower back pain. However, both drugs reported severe adverse reactions in joints, including osteonecrosis and rapidly progressive osteoarthritis (RPOA), (Jayabalan and Schnitzer 2016). Therefore, it is of great social significance and clinical prospect to develop safer anti-NGF antibodies, in order to meet clinical needs and improve patients’ quality of life.

DS002 is an anti-NGF monoclonal antibody independently developed by Dartsbio Pharmaceuticals, Ltd. *In vitro* studies have shown that DS002 can successfully block the binding of NGF to TrkA receptors. In pre-clinical studies, DS002 treatment demostrated potent analgesic effects in several pain models including OA pain, chemotherapy-induced pain, and cancer pain. Based on the preclinical repeated toxicity test and efficacy test, as well as the clinical trial of drugs at the same target (Jonsson et al., 2016), the dose range selection of this first-in-human trial for the healthy subject was set as 0.5 mg–20.0 mg. The objective of this first-in-human study was to evaluate the safety, tolerability, pharmacokinetics and immunogenicity of a single subcutaneous injection of DS002 in healthy volunteers.

## Methods

This study (CTR20210155) was approved by the ethics committee of Nanjing Drum Tower Hospital, The Affiliated Hospital of Nanjing University Medical School, and carried out according to the Declaration of Helsinki (2013 version), the Guideline For Good Clinical Practice issued by the International Council for Harmonisation (ICH), the Good Clinical Practice (GCP) issued by the National Medical Products Administration (NMPA) and relevant regulations. Written informed consent was obtained from all subjects.

### Study design

The overall study was a single-center, randomized, double-blind, single-dose escalation, placebo-controlled phase I clinical trial. A total of 53 Chinese healthy subjects were enrolled in this study, including 27 males and 26 females, and 52 subjects completed this clinical trial.

**FIGURE 1:**
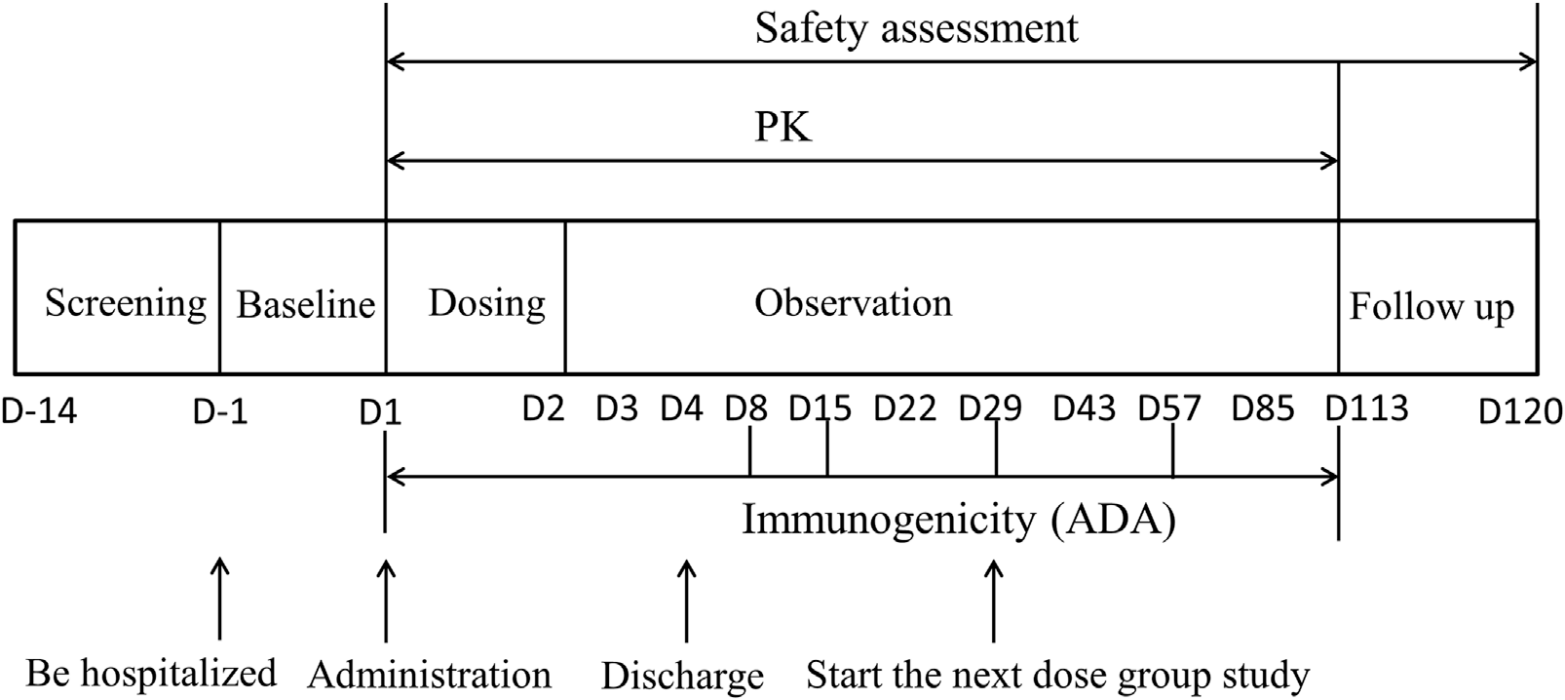
The flow chart of DS002 single-dose administration. PK, pharmacokinetics; ADA, anti-drug antibody.

The dose-escalation design was used to evaluate the safety, tolerability, pharmacokinetics and immunogenicity of DS002 after a single administration. Seven dose groups were set up, including 0.5, 1.0, 2.0, 4.0, 7.0, 12.0 and 20.0 mg, not per kg, respectively. DS002 was administered by subcutaneous injection. Four subjects were enrolled in the first dose group (0.5 mg), and each subject received a singledose of DS002 subcutaneously. In the 2nd to 7th dose groups, eight subjects were enrolled in each group, among whom 6 received DS002 and two subjects received placebo. A series of blood samples were collected before and after DS002 administration to evaluate the PK parameters and immunogenicity of DS002 in healthy subjects. Subjects were clinically followed up on the 4th, 8th, 15th, 22nd, 29th, 43rd, 57th, 85th and 113th days, biological samples were collected and safety and tolerability were assesseded. Subjects were followed up by telephone on the 120th day, which was the end of each dose group. (Figure 1). The next dose escalation group could only be started after the assessment results on the 29th day of all subjects in the previous dose group were confirmed as safe.

During hospitalization in the research center, the subjects were managed with a unified diet and rest protocol. On the day before DS002 administration, the subjects stayed in the Phase I trials unit, took a unified dinner, and then fasted overnight for at least 10 h. The next morning, they ate a small amount of standard breakfast and then received DS002 injection subcutaneously. After injection, they ate the standard meals. Within 48 h prior to enrollment and during the whole trial period, it was forbidden to take any food and drink rich in caffeine or xanthine (such as tea, coffee, chocolate, cola, etc.), smoking and drinking alcohol-containing products were also not allowed. Strenuous exercise (exceeding normal daily activities) were avoided during the whole trial period. Subjects were told to take proper contraception from the start of the study period to 6 months after DS002 administration.

This study was a double-blind trial design (except for the first dose group). Blindness was maintained for the generation of random numbers, the coding of drugs used in the trial, the drug matched to each subject, the recording and evaluation of test results, the monitoring of the test process and data management. On the day of DS002 administration, the investigator checked the information of all subjects to ensure that they met the inclusion criteria of the trial, and then administered according to the drug number matched with the random number of the subject. All relevant personnel remained blind during the clinical study.

PK blood sampling time points: for determination of the concentration of DS002, blood samples were collected at 17 time points in each dose group: within 1 h before DS002 administration, and 6 h, 8 h, 12 h, D2 (24 h), D2 (36 h), D3 (48 h), D3 (60 h), D4 (72 h), D8 (168 h), D15 (336 h), D22 (504 h), D29 (672 h), D43 (1,008 h), D57 (1,344 h), D85 (2016 h), and D113 (2688 h) after administration. About 3 ml of venous blood was collected at each time point (Figure 1).

Immunogenicity (ADA) blood sampling time points: blood samples were collected at 6 time points in each dose group, including within 1 h before administration, D8 (168 h), D15 (336 h), D29 (672 h), D57 (1,344 h) and D113 (2688 h) after administration, and about 3 ml of venous blood was collected at each time point (Figure 1).

### Subjects

Subjects were healthy volunteers aged 18–45 years, including both males and females (not pregnant or breastfeeding). The body mass index (BMI) of the subjects was 19.0–26.0 kg/m^2^, the male body weight was ≥50 kg and female ≥45 kg. Effective contraception should be used during the trial and continued for 6 months after administration. Subjects fully understood the content, objectives and characteristics of the study and could complete the study as planned, voluntarily participated in the study and signed written informed consent.

Subjects were excluded if they met one of the following: Those who had an allergic history or allergic constitution in the past; Those who had a history of bone or joint diseases, peripheral or autonomic neuropathy, spontaneous bleeding of unknown causes and (or) disease or medical history of continuous bleeding after trauma, abnormal thyroid function or thyroid hormone, or malignant tumor; Smokers, alcoholics or drug abusers. Subjects were not allowed to take any prescription drugs, over-the-counter drugs and natural health products within 14 days before screening.

### Objectives

The primary objective of this study was to evaluate the safety and tolerability of DS002 as a single administration in healthy subjects. The secondary objective was to evaluate the pharmacokinetic parameters and immunogenicity of DS002 in healthy subjects after a single administration.

### Research indicators

The safety indicators of this study included the incidence of adverse event (AE, Graded according to CTCAE v5.0) and serious adverse event (SAE); Laboratory examinations; Neurological examination; Injection site reaction; 12-lead electrocardiogram (ECG); Abdominal B ultrasound; Vital signs; Physical examination.

PK indexes: PK parameters of DS002 after a single administration, including but not limited to C_max_, T_max_, AUC_0-t_, AUC_0-inf_, t_1/2_, MRT, λ_z_; Immunogenicity index: ADA after single administration of DS002.

### The methods for detecting DS002 and anti-DS002 antibody

Electrochemiluminescence Immunoassay (ECLIA) methods for the analysis of the pharmacokinetics (PK) and anti-drug antibody (ADA) of DS002 in serum were established.

For PK samples, the MSD plate was pre-coated with huNGF-his, and after adding the sample, it was incubated to form an antigen-antibody complex. After incubation, the unbound DS002 was washed away with a washing solution. Detection antibody Sulfo-Anti-DS002 antibody was then added and incubated; after incubation, unbound antibody was washed off with washing solution; finally the reading buffer was added, and MSD (MESO QuickPlexSQ120, acquisition software MSD Workbench V4.0.12) was used to collect the signal. The data were analyzed and processed with Watson LIMS (V7.4.1 and V7.6.1). The calibration ranges of DS002 were 2.50 ng/ml∼500.00 ng/ml. The quality control samples (7.5, 50 and 400 ng/ml) were determined with an accuracy of 89.0%–114.3%, the precision of 17.6% (CV) for inter-assay and accuracy of 104.0%–106.1%, precision of 19.8% (CV) for intra-assay. There was no matrix effect for human serum. No hook effect was observed for samples up to 5000.00 ng/ml. The sample can be diluted up to 1,250 fold with a total CV(%) of 5.5%.

The ADA samples were measured by screening assay, confirmatory assay, and titer assay. The method is based on the multivalency of antibodies and the specific binding of antigen-antibody. First, each control sample and the test sample were diluted by 1:20 times of acidification. Then each diluted sample solution was added to TrkA-hFc working solution for purification to remove interferences. The purified solution was then added with Biotin-DS002 and Suflo-DS002 Master Mixture (with or without drug), incubating on a dilution plate to allow a bridging reaction between the drug and the anti-drug antibody to form a bridging complex. After the reaction was completed, it was transferred to the MSD plate for incubation. After incubation, the plate was washed and then the reading buffer was added, and the signal was read on the MSD. For the screening assay, the HPC samples (5000 ng/ml) were determined with a precision of 4.4% (CV) for inter-assay and 11.9% (CV) for intra-assay. The LPC samples (16 ng/ml) were determined with a precision of 14% (CV) for inter-assay and 10.8% (CV) for intra-assay. For the confirmatory assay, the HPC samples (5000 ng/ml) were determined with a precision of 0.1% (CV) for inter-assay and 0.1% (CV) for intra-assay. The LPC samples (16 ng/ml) were determined with a precision of 25.9% (CV) for inter-assay and 18.3% (CV) for intra-assay. There was no matrix effect for human serum. No hook effect was observed for samples up to 41000.00 ng/ml.

### Data analysis and statistical methods

Unless otherwise specified, SAS (version 9.4 and above) and Phoenix™ WinNonlin ^®^ (Version 8.0 and above) were used for statistical analysis. The classified variables were summarized by frequency and percentage. Continuous variables were summarized by sample size, mean, standard deviation, median, minimum and maximum. For some continuous variables, the coefficient of variation or the geometric coefficient of variation, geometric mean, lower quartile and upper quartile were also counted. The number of subjects enrolled in each dose group and the situation of dropped cases were summarized. A descriptive statistical analysis on baseline characteristics of the enrolled cases were made. Safety and PK data were summarized using appropriate statistical tables and diagrams.

Full analysis set (FAS): all randomized subjects. Safety analysis set (SS): all subjects who have received the study drug at least once and have at least one safety evaluation index. PK concentration and parameter analysis set (PKCS and PKPS): all subjects who have used the study drug and have at least one PK concentration or parameter data. Immunogenicity analysis set (IS): all subjects who have received the study drug at least once and have at least one post-baseline immunogenicity evaluation data.

Based on the PKCS, the concentration data were tabulated and described by dose groups. The drug concentration-time curves (linear and semi-logarithmic) were drawn according to the planned blood collection time.

Based on PKPS, a Non-Compartmental Analysis by Phoenix™ WinNonlin^®^ (version 8.0 or above) was adopted. PK parameters were calculated according to the actual blood collection time, and PK parameters were listed and described statistically. A power model was used to analyze the linear relationship between main PK parameters (C_max_, AUC) and dose.

## Results

### Subject demographics

**FIGURE 2:**
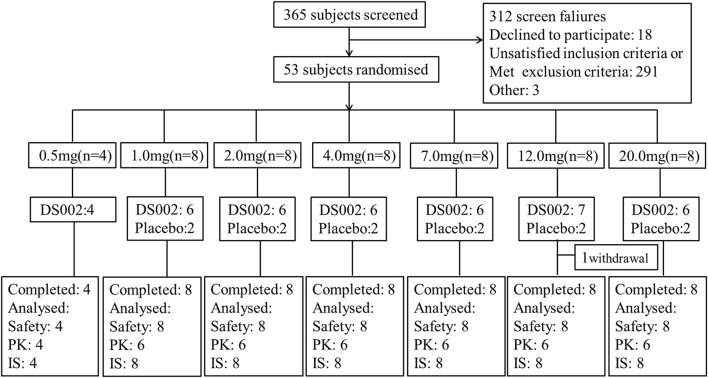
Subject disposition of participants in the study. PK, pharmacokinetics; IS, Immunogenicity analysis set.

The study was conducted from 22 February 2021 to 24 May 2022. In this trial, a total of 53 healthy Chinese subjects in seven dose groups were included in the FAS, and 52 were included in the SS (one subject withdrew from the study before administration), including 40 subjects receiving DS002, four in the 0.5 mg dose group, and six in other dose groups; There were 12 subjects receiving placebo. All 52 subjects completed the study (Figure 2). The demographics and baseline characteristics analysis of the subjects are shown in Table 1.

**TABLE 1:** Subject demographics.

	0.5 mg *n* = 4	1.0 mg *n* = 6	2.0 mg *n* = 6	4.0 mg *n* = 6	7.0 mg *n* = 6	12.0 mg *n* = 7	20.0 mg *n* = 6	Placebo *n* = 12	Total *n* = 52
Age, years	27.00 (2.16)	31.00 (5.87)	28.33 (8.21)	26.83 (8.93)	31.00 (7.95)	28.86 (8.69)	29.33 (5.61)	27.83 (6.24)	28.74 (6.77)
Female, n (%)	1 (25.0)	2 (33.3)	3 (50.0)	4 (66.7)	5 (83.3)	3 (42.86)	3 (50.0)	5 (41.7)	26 (49.06)
Male, n (%)	3 (75.0)	4 (66.7)	3 (50.0)	2 (33.3)	1 (16.7)	4 (57.14)	3 (50.0)	7 (58.3)	27 (50.94)
Han, n (%)	4 (100)	6 (100)	6 (100)	6 (100)	6 (100)	7 (100)	5 (83.33)	12 (100)	52 (98.11)
Height, cm	165.13 (3.35)	164.25 (11.23)	164.33 (6.84)	164.33 (6.46)	162.58 (8.65)	163.21 (8.35)	164.67 (10.60)	165.88 (8.26)	164.42 (7.93)
Weight, kg	64.68 (5.19)	62.60 (10.84)	63.92 (8.62)	56.15 (3.89)	57.78 (6.91)	59.16 (5.04)	61.57 (7.67)	62.43 (9.90)	61.02 (7.91)
BMI, kg/m^2^	23.68 (1.13)	23.08 (2.05)	23.58 (1.95)	20.83 (1.71)	21.83 (1.35)	22.21 (1.38)	22.72 (2.23)	22.60 (2.12)	22.52 (1.90)

Age and BMI are presented as mean (standard deviation). BMI, body mass index; Han, Han Nationality.

### Safety and tolerability

At least one adverse event (AE) occurred in 36 of 40 (90%) healthy subjects who received DS002 and 12 of 12 (100%) subjects who received placebo. Forty-five subjects (86.54%) had adverse reactions (ARs). 12 (25.00%) subjects of DS002 had AEs of grade 2 (vs. placebo, 1/12, 8.33%). No grade 3 or grade 4 AEs were reported. The TEAEs of subjects are shown in Table 2.

**TABLE 2:** Summary of adverse events.

n (%)	0.5 mg *n* = 4		1.0 mg *n* = 6	2.0 mg *n* = 6		4.0 mg *n* = 6	7.0 mg *n* = 6	12.0 mg *n* = 6	20.0 mg *n* = 6	Placebo *n* = 12	Total *n* = 52
All AEs	3 (75.00)		5 (83.33)	5 (83.33)		5 (83.33)	6 (100)	6 (100)	6 (100)	12 (100)	48 (92.31)
All ARs	2 (50.00)		4 (66.67)	5 (83.33)		4 (66.67)	6 (100)	6 (100)	6 (100)	12 (100)	45 (86.54)
Grade 2 AEs	1 (25.00)		1 (16.67)	0		3 (50.00)	3 (50.00)	2 (33.33)	2 (33.33)	1 (8.33)	13 (25.00)
Grade 2 ARs	0		0	0		0	0	1 (16.67)	0	0	1 (1.92)
Term											
Various inspections											
Elevated AMY	1 (25.00)		1 (16.67)	1 (16.67)		1 (16.67)	1 (16.67)	2 (33.33)	1 (16.67)	4 (33.33)	12 (23.08)
Reduced T3	0		1 (16.67)	2 (33.33)		0	3 (50.00)	0	3 (50.00)	2 (16.67)	11 (21.15)
Elevated UA	1 (25.00)		1 (16.67)	3 (50.00)		1 (16.67)	1 (16.67)	0	2 (33.33)	1 (8.33)	10 (19.23)
Elevated TSH	0		0	1 (16.67)		1 (16.67)	1 (16.67)	2 (33.33)	1 (16.67)	3 (25.00)	9 (17.31)
Decreased WBC	0		0	0		0	1 (16.67)	2 (33.33)	2 (33.33)	3 (25.00)	8 (15.38)
Increased N	0		0	1 (16.67)		0	0	0	1 (16.67)	3 (25.00)	5 (9.62)
Elevated ALT	0		0	1 (16.67)		0	0	2 (33.33)	0	1 (8.33)	4 (7.69)
Urine RBC(+)	0		0	0		1 (16.67)	1 (16.67)	0	1 (16.67)	1 (8.33)	4 (7.69)
Urine OB(+)	0		0	0		1 (16.67)	1 (16.67)	0	1 (16.67)	1 (8.33)	4 (7.69)
Abnormal ECG T wave	0		0	0		2 (33.33)	0	0	0	2 (16.67)	4 (7.69)
Elevated TG	0		0	1 (16.67)		0	1 (16.67)	1 (16.67)	0	1 (8.33)	4 (7.69)
Increased WBC	0		0	0		1 (16.67)	0	0	1 (16.67)	1 (8.33)	3 (5.77)
Prolonged QTc	0		2 (33.33)	0		0	0	0	1 (16.67)	0	3 (5.77)
Hypokalemia	0		1 (16.67)	0		0	0	1 (16.67)	1 (16.67)	0	3 (5.77)
Decreased FT4	0		1 (16.67)	1 (16.67)		0	1 (16.67)	0	0	0	3 (5.77)
Elevated FT4	0		0	0		1 (16.67)	0	1 (16.67)	0	1 (8.33)	3 (5.77)
Elevated γ-GT	0		0	1 (16.67)		0	0	1 (16.67)	0	0	2 (3.85)
Decreased T4	0		0	0		0	0	0	1 (16.67)	1 (8.33)	2 (3.85)
Urine protein (+)	0		0	0		0	0	0	0	2 (16.67)	2 (3.85)
Elevated AST	0		0	1 (16.67)		0	0	1 (16.67)	0	0	2 (3.85)
Elevated TBil	0		0	1 (16.67)		0	0	0	0	1 (8.33)	2 (3.85)
Elevated Ca	0		0	0		2 (33.33)	0	0	0	0	2 (3.85)
Elevated FT3	1 (25.00)		0	1 (16.67)		0	0	0	0	0	2 (3.85)
Abnormal liver ultrasound	0		0	1 (16.67)		0	0	0	0	0	1 (1.92)
Decreased L	0		0	0		0	0	0	0	1 (8.33)	1 (1.92)
Elevated FT3	0		0	0		0	0	0	1 (16.67)	0	1 (1.92)
Increased Eos	0		0	0		0	1 (16.67)	0	0	0	1 (1.92)
Increased heart rate	0		0	0		1 (16.67)	0	0	0	0	1 (1.92)
Decreased Ca	0		0	0		0	0	0	0	1 (8.33)	1 (1.92)
Decreased Hb	0		0	0		0	1 (16.67)	0	0	0	1 (1.92)
Decreased P	0		0	0		0	0	1 (16.67)	0	0	1 (1.92)
Elevated Glu	0		0	1 (16.67)		0	0	0	0	0	1 (1.92)
Decreased FT3	0		0	1 (16.67)		0	0	0	0	0	1 (1.92)
Increased N%	0		0	0		0	0	0	0	1 (8.33)	1 (1.92)
Infections and infectious diseases											
URTI	1 (25.00)		0	0		1 (16.67)	4 (66.67)	1 (16.67)	3 (50.00)	0	10 (19.23)
Urinary tract infection	0		0	0		0	0	0	0	1 (8.33)	1 (1.92)
Vaginal fungal infection	0		0	0		0	1 (16.67)	0	0	0	1 (1.92)
Pharyngitis	0		1 (16.67)	0		0	0	0	0	0	1 (1.92)
Tinea pedis	0		0	0		0	1 (16.67)	0	0	0	1 (1.92)
Various musculoskeletal and connective tissue diseases											
Arthralgia	0		0	0		1 (16.67)	0	1 (16.67)	2 (33.33)	1 (8.33)	5 (9.62)
Joint swelling	0		0	0		0	0	0	1 (16.67)	0	1 (1.92)
Diseases of hepatobiliary system											
Gallbladder polyps	1 (25.00)		0	0		0	0	0	0	1 (8.33)	2 (3.85)
Hepatic steatosis	0		0	1 (16.67)		0	0	0	0	0	1 (1.92)
Systemic diseases and various reactions at the administration site											
Bleeding	0		0	0		0	0	0	0	1 (8.33)	1 (1.92)
Erythema	0		0	0		1 (16.67)	0	0	0	0	1 (1.92)
Metabolic and nutritional diseases											
Hypoglycemia	0		0	0		0	0	0	1 (16.67)	0	1 (1.92)
Skin and subcutaneous tissue diseases											
Urticaria	0		0	0		1 (16.67)	0	0	0	0	1 (1.92)
Heart Organ Diseases											
Ventricular extrasystole	0		1 (16.67)	0		0	0	0	0	0	1 (1.92)
Eye organ diseases											
Keratitis	0		0	0		1 (16.67)	0	0	0	0	1 (1.92)

AEs, adverse events; AR, adverse reactions, the adverse events that are positively related, likely related, and possibly related. AMY, amylase; T3, triiodothyronine; UA, serum uric acid; TSH, serum thyrotropin; WBC, white blood cell count; N, neutrophil count; ALT, alanine aminotransferase; RBC, red blood cell; OB(+), occult blood positive; TG, triglycerides; QTc, QTc interval of ECG; FT4, free thyroxine; GT, glutamyltransferase; T4, thyroxine; AST, aspartate aminotransferase; TBil, total bilirubin; Ca, serum calcium; FT3, free triiodothyronine; L, lymphocyte count; Eos, eosinophil count; Hb, Hemoglobin; P, serum phosphorus; Glu, serum glucose; URTI, upper respiratory tract infection. There were no serious adverse events during the entire study and no TEAEs that led to treatment discontinuation or interruption.

Among the 40 subjects injected DS002, the AEs with an incidence rate of more than 5% include the following: upper respiratory tract infection (25.00%, 10/40); decreased triiodothyronine, and elevated serum uric acid (22.50%, 9/40); elevated amylase (20.00%, 8/40); elevated serum thyrotropin (15.00%, 6/40); decreased white blood cell count (12.50%, 5/40); arthralgia (10.00%, 4/40); positive urine red blood cell, positive urine occult blood, elevated triglycerides, elevated alanine aminotransferase, prolonged QTc interval of ECG, hypokalemia, and decreased free thyroxine (7.50%, 3/40); increased neutrophil count, abnormal ECG T wave, increased white blood cell count, elevated free thyroxine, elevated γ-glutamyltransferase, elevated aspartate aminotransferase, elevated serum calcium and elevated free triiodothyronine (5.00%, 2/40).

In the placebo group, 12 subjects (100.00%) had AEs. The incidence rates from high to low were elevated amylase (33.33%, 4/12); elevated serum thyrotropin, decreased leukocyte count, and increased neutrophil count (25.00%, 3/12); decreased triiodothyronine, abnormal ECG T wave, and positive urine protein (16.67%, 2/12); all (100.00%) had ARs. Of which one subject (8.33%) had 2 times Grade 2 AE.

The incidences of AEs and ARs in each dose group showed an overall upward trend with dose escalation, and the incidence of AEs and ARs between DS002 and placebo subjects was similar. One case of Grade 2 AE (elevated amylase without symptom) was reported in the 12.0 mg dose group, whilst the rest of elevated amylase AEs were all Grade 1. No serious adverse event (SAE) was reported in subjects who received DS002 injection. None of the subjects died or dropped out to TEAE.

In brief, after a single dose of DS002, none of the dose groups reached the termination standard of dose escalation, and the safety and tolerability profiles of DS002 were satisfied.

### Pharmacokinetics

All 40 subjects receiving DS002 were included in the PK analysis. The PK parameters of the DS002 and placebo groups are shown in Table 3.

**TABLE 3:** The pharmacokinetic parameters of DS002.

	0.5 mg *n* = 4	1.0 mg *n* = 6	2.0 mg *n* = 6	4.0 mg *n* = 6	7.0 mg *n* = 6	12.0 mg *n* = 6	20.0 mg *n* = 6
T_max_ (h)	167.77 (48.00–335.78)	168.63 (168.58–336.25)	251.90 (35.93–335.88)	168.54 (168.50–504.58)	337.38 (35.95–337.48)	168.61 (168.58–337.82)	169.61 (59.50–337.35)
C_max_ (ng/ml)	51.83 ± 22.74	85.69 ± 37.80	157.92 ± 63.52	295.97 ± 69.58	374.02 ± 42.75	956.21 ± 213.41	2048.86 ± 564.78
t_1/2_(h)	176.80 ± 66.67	205.46 ± 55.76	235.16 ± 48.83	214.06 ± 29.67	223.92 ± 39.93	294.23 ± 24.17	287.18 ± 45.24
AUC_0-t_ (h·ng/mL)	20615.16 ± 5698.28	48976.41 ± 13712.08	107834.02 ± 49871.96	238871.15 ± 62280.97	332402.49 ± 84014.77	717276.99 ± 113683.18	1669608.11 ± 387246.36
AUC_0-inf_ (h·ng/mL)	21852.45 ± 5920.21	50946.19 ± 13253.08	112353.93 ± 48514.62	242206.02 ± 61448.70	336848.83 ± 84030.42	726968.53 ± 112493.19	1673504.66 ± 389106.13
λ_z_ (1/h)	0.0043 ± 0.0012	0.0036 ± 0.0009	0.0031 ± 0.0007	0.00 ± 0.00	0.0033 ± 0.0005	0.0032 ± 0.0006	0.0024 ± 0.0002
CL (mL/h)	24.75 ± 9.18	20.99 ± 6.49	20.53 ± 8.28	17.45 ± 4.55	21.89 ± 5.42	16.87 ± 2.86	12.69 ± 3.91
V_z_ (mL)	6119.27 ± 2314.67	6517.12 ± 3671.56	6851.26 ± 2815.53	5365.67 ± 1,437.83	6967.68 ± 1778.05	7212.00 ± 1,618.54	5265.42 ± 1896.20
MRT_0-t_ (h)	297.91 ± 126.91	398.31 ± 83.78	457.03 ± 98.71	519.61 ± 107.44	556.17 ± 126.16	557.95 ± 54.15	589.40 ± 46.42
MRT_0-inf_ (h)	343.10 ± 139.90	443.53 ± 108.34	505.59 ± 103.18	539.77 ± 105.51	576.68 ± 126.44	583.91 ± 62.09	595.14 ± 49.79
AUC__%Extrap_ (%)	5.81 ± 1.09	4.42 ± 3.59	4.66 ± 5.41	1.50 ± 1.21	1.41 ± 1.26	1.38 ± 0.47	0.23 ± 0.17

T_max_ as median (range); C_max_, t_1/2,_ AUC_0-t_, AUC_0-inf_, λ_z_, CL, V_z_, MRT_0-t_ and MRT_0-inf_ are presented as mean ± standard deviation. T_max_, time to reach maximum concentration; C_max_, maximum concentration; t_1/2_, elimination half-life; AUC_0-t_, area under the plasma concentration-time curve from time 0 to last time of quantifiable concentration; AUC_0-inf_, area under the plasma concentration-time curve from time 0 extrapolated to infinite time; λ_z_, elimination rate constant; CL, clearance rate; V_z_, apparent volume of distribution; MRT_0-t_, mean residence time from time 0 to last time of quantifiable concentration; MRT_0-inf_, mean residence time from time 0 extrapolated to infinite time. AUC__% Extrap_ values are calculated using the percentage of AUC_0-t_ and AUC_0-inf_ values (100%–AUC_0-t_%/AUC_0-inf_).

In this study, AUC__% Extrap_ of DS002 in the dose range of 0.5 mg–20 mg was less than 5.81% (Table 3), which indicated that the design of the blood sampling time points for PK test was reasonable, and the calculated terminal clearance rate constant and related parameters were reliable.

**FIGURE 3:**
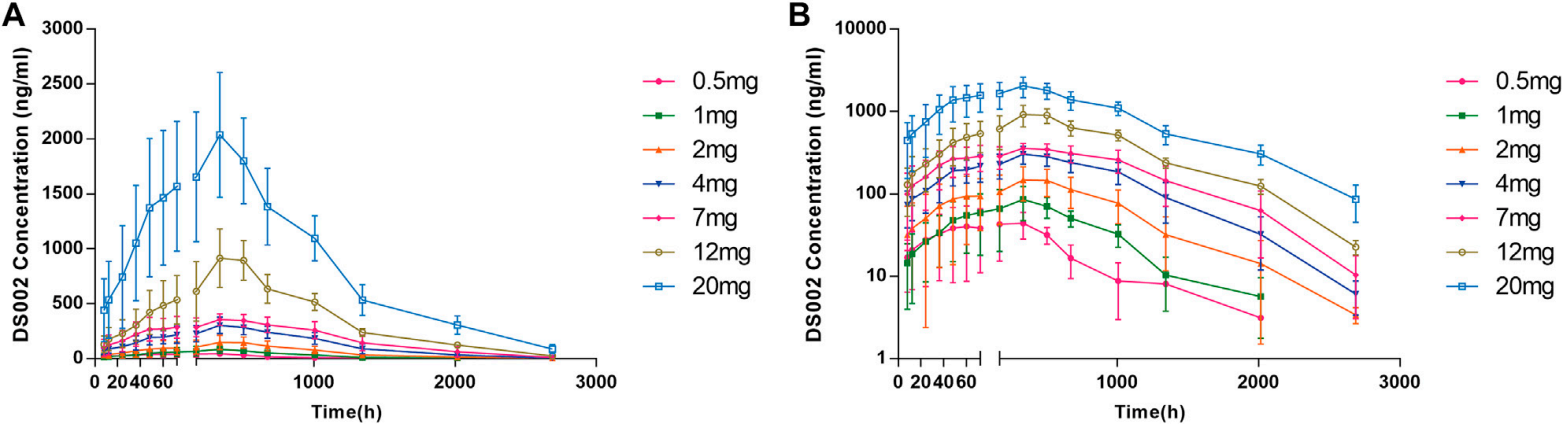
Plasma concentration-time profile (PKCS) of DS002. **(A)** DS002 concentrations time curves of subjects; **(B)** Semi-logarithmic curves of DS002 concentrations time of subjects. PKCS, pharmacokinetics concentration set. Data are presented as mean ± SD.

After a single subcutaneous injection of DS002 in healthy subjects, the median T_max_ was 167.77–337.38 h; The mean t_1/2_ was 176.80–294.23 h. Except for the 0.5 mg dose group, of which t_1/2_ was 176.80 h, t_1/2_ of the remaining dose groups ranged 205.46–294.23 h (Table 3). In the dose range of 0.5–20.0 mg, C_max_, AUC_0-t_ and AUC_0-inf_ of prototype drug DS002 increased with the increase of dose (Table 3; Figures 3A,B).

After a single DS002 administration to healthy subjects, C_max_, AUC_0-t_, AUC_0-inf_ and dose were simultaneously logarithmically converted to perform linear PK analysis. The 90% confidence interval of C_max_ slope was 90.61%–106.92%, the 90% confidence interval of AUC_0-t_ slope was 107.41%–121.13%, and the 90% confidence interval of AUC_0-inf_ slope was 106.05%–119.33%. The results indicated that C_max_ and AUC_0-inf_ increased with dose, but did not have a significant dose-linear relationship, whilst AUC0-t was not dose dependent at all. (Table 4).

**TABLE 4:** DS002 Linear pharmacokinetic analysis of indicators (C_max_, AUC_0-t_, AUC_0-inf_).

Parameters	Dose range (mg)	Interval (%)	Regression equation	Slope		Linear relationship
				Point estimate (%)	90%CI (%)	
C_max_(ng/mL)	0.5–20.0	93.95–106.49	Y = 0.99*x+4.35	98.77	(90.61,106.92)	Unidentified
AUC_0-t_ (ng·h/mL)	0.5–20.0	93.95–106.49	Y = 1.14*x+10.70	114.27	(107.41,121.13)	No
AUC_0-inf_ (ng·h/mL)	0.5–20.0	93.95–106.49	Y = 1.13*x+10.75	112.69	(106.05,119.33)	Unidentified

C_max_, maximum concentration; AUC_0-t_, area under the plasma concentration-time curve from time 0 to last time of quantifiable concentration; AUC_0-inf_, area under the plasma concentration-time curve from time 0 extrapolated to infinite time. CI, confidence interval.

### Immunogenicity

The ADA test results of every group of subjects in this trial were negative at all follow-up visits, indicating that the immunogenicity of the DS002 injection was low.

## Discussion

Pain can be treated more effectively with analgesics that operate on different mechanisms. Traditional NSAIDs and opioids are the most widely used analgesics, but their long-term use causes side effects and addiction, which is unable to meet clinical needs (Chang et al., 2016; Coussens et al., 2019; Kiyatkin 2019; Schjerning et al., 2020). DS002, an anti-NGF monoclonal antibody, is developed as a potential analgesic. Preclinical studies have shown that it could effectively supress NGF/TrkA signalingand reset the pain threshold without obvious adverse reactions and addiction problems. Here in this first-in-human study, we evaluated the safety, tolerability and PK parameters of DS002 as a single subcutaneous injection in healthy subjects. DS002 was well tolerated and safe within the dose range of 0.5–20.0 mg. PK results showed that the absorption and metabolism of DS002 was slow, and had a long effect time. Immunogenicity results suggested that ADA was negative throughout all follow-up visits.

In terms of safety, all subjects receivied DS002 injection completed the trial. No dose-limiting toxicity was observed, and the dose increment termination criteria were not reached within the dose range of 0.5–20 mg. Five subjects suffered from arthralgia, including four subjects receiving DS002 (one in 4.0 mg, one in 12.0 mg and two in 20.0 mg group) and one subject in the placebo group. The symptoms were mild without any discomfort such as activity disorder, and were all resolved without intervention. It is worth noting that no severe bone and joint events or peripheral neuropathy/paresthesia were found. No rapid progression of osteoarthritis (RPOA) was observed even at high doses, which was the most prominent concern when the US Food and Drug Administration shelved the development plan of similar anti-NGF drugs. Collectively, the safety data of DS002 indicate that it may serve as provide a new therapeutic strategy for the target of analgesic treatment.

In adults, NGF and its interaction with TrkA have been found to play a vital role in the nociception and plasticity of the nervous system under pain conditions. Therefore, various monoclonal antibody therapies targeting this pathway have been studied to treat chronic pain. Although the Food and Drug Administration once shelved the development plan for an anti-NGF monoclonal antibody due to safety concerns, this target still has great clinical value and application prospect. Currently, no anti-NGF monoclonal antibody has been approved for clinical use, but three anti-NGF drugs are under development: tanezumab, fulranumab and fasinumab (Enomoto et al., 2019; Zhao et al., 2022). Tanezumab was designated as a fast track (Hochberg et al., 2016). The monoclonal antibody has unique pharmacokinetic parameters such as non-linear pharmacokinetics metabolism, time dependence and long half-life; Target-mediated distribution (TMD) and clearance (TMC) are important pathways for macromolecular drug metabolism. The occurrence of TMD and TMC are the important sources of non-linear PK characteristics. The t_1/2_ of tanezumab is about 21 day (Jonsson et al., 2016), while that of DS002 was around 7–12 days. In the dose range of 0.5–20.0 mg, C_max_, AUC_0-t_ and AUC_0-inf_ of prototype drug DS002 increased with the increase in dosage. C_max_ and AUC_0-inf_ did not show a significant dose linear relationship, whilst AUC_0-t_ was not dose dependent at all. The PK parameters of DS002 conform to the metabolic characteristics of monoclonal antibodies. Moreover, the immunogenicity of DS002 injection was low (ADA was negative).

In conclusion, the absorption and metabolism of a single subcutaneous injection of DS002 in healthy subjects were slow, it exhibited a low volume of distribution, and the clearance rate was low. Single subcutaneous injection of DS002 was well tolerated and non-immunogenic within the dose range of 0.5 mg–20.0 mg. The anti-NFG antibody represented by DS002 shows promise as a novel, safe and non-addictive approach for pain treatment. This first-in-human study supports further development of DS002 as a potential analgesic treatment.

## Data Availability

The original contributions presented in the study are included in the article/Supplementary Material, further inquiries can be directed to the corresponding authors.
